# Increase in age at onset of moyamoya disease in China over 25 years

**DOI:** 10.1002/brb3.3034

**Published:** 2023-05-07

**Authors:** Zhiyang Ma, Dayu Chen, Sheng Wang, Yaozu Zhu, Jincao Chen

**Affiliations:** ^1^ Department of Neurosurgery Xijing Hospital of Air Force Military Medical University Xi'an China; ^2^ Department of Neurosurgery Zhongnan Hospital of Wuhan University Wuhan China; ^3^ Department of Neurosurgery Wuhan General Hospital of Guangzhou Military Command Wuhan China; ^4^ Department of Neurosurgery, Tongji Medical College Huazhong University of Science and Technology Wuhan China; ^5^ Department of Neurosurgery Xiangyang Central Hospital Xiangyang China

**Keywords:** age at onset, birth cohort, etiology, leptospiral exposure, moyamoya disease

## Abstract

**Background:**

To explore whether the age at onset (AAO) of Chinese patients with moyamoya disease (MMD) increased over time due to a reduced exposure to leptospiral infection.

**Methods:**

We performed an independent, multicenter, retrospective study based on data from patients with MMD who initially attended four tertiary hospitals in Hubei, China, from 1996 to 2020. After stratifying the year of MMD onset into five periods (1996–2000, 2001–2005, 2006–2010, 2011–2015, and 2016–2020), we analyzed the temporal trends in AAO and compared different classes of AAO (early‐onset, < 20 years; intermediate‐onset, 20–49 years; late‐onset, ≥ 50 years) in each period.

**Results:**

We included 1858 patients in this study, with 878 women and 980 men. Their median (IQR) AAO was 47 (39‒55) years. The case AAO significantly increased at the rate of 0.94 years per year (*r* = 0.406, *p* < .0001), while no trend was observed in birth years through time (*p* = .512). The birth cohorts who grew up in the leptospirosis epidemic years was stably susceptible to MMD. The median (IQR) AAO has increased significantly from 26 (14–37) years (1996–2000) to 51 (43–57) years (2016–2020) (*p* < .0001). The proportion of early‐onset MMD was significantly higher in 1996–2000 (33.3%, *p* < .0001) and 2001–2005 (10.4%, *p* < .001). The AAO shows an aging trend that the proportion of late‐onset MMD went from 4.5% (2001–2005) to 54.5% (2016–2020) (*p* < .0001).

**Conclusions:**

The AAO of MMD was increasing during a recent 25‐year period in China, which may reflect a birth cohort effect that resulted from environmental changes. The disparity risk of birth cohorts with MMD changed with leptospirosis epidemics, suggesting leptospiral exposure might be a potential risk factor.

## Graphical abstract

In this article, we found increased age at onset of moyamoya disease over a 25‐year period, which reflect a birth cohort effect.

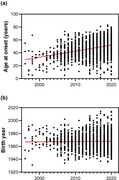

## 1 INTRODUCTION

Moyamoya disease (MMD), a cerebrovascular disorder characterized by chronic progressive occlusion of the terminal internal carotid artery and dilated collateral microvessels near the base skull, developing collateral circulation like “puff of smoke” on angiography (Suzuki & Takaku, 1969). MMD is a leading cause of stroke for both children and young adult, while the etiology remains largely unclear. Genetic studies have identified RNF213, as a susceptibility gene for MMD, but the low penetrance in genetically susceptible individuals suggests that a second hit is necessary to trigger disease onset (Asselman et al., 2022).

In the second half of the 20th century, leptospirosis severely threatened public health in China. During this period, a unique neurologic disorder named leptospiral cerebral arteritis was broadly reported over China, which interestingly presented typical angiographic and pathological features of MMD (Liu et al., 1980; Liu et al., 1978). Recently, epidemiologic studies from China uncovered the onset of MMD shows a clustered regional pattern, and leptospirosis may explain a cluster observed in Hubei province (Ma et al., 2021; Zhang et al., 2022). These findings suggested a causal link might exist between leptospiral exposure and MMD.

**FIGURE 1:**
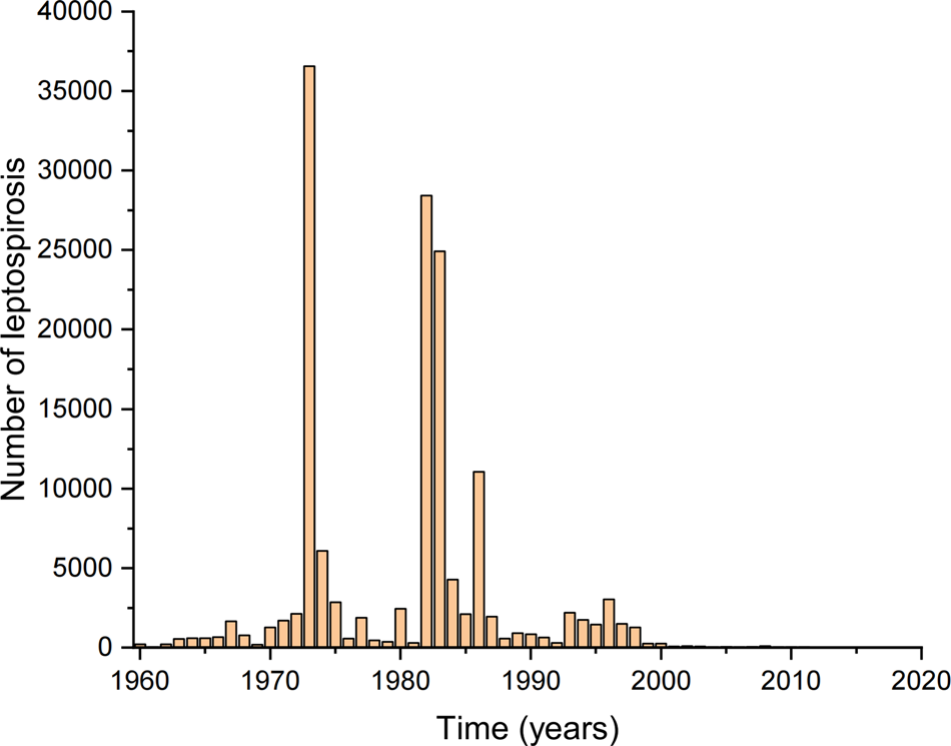
The annual surveillance data of leptospirosis in Hubei province (1960–2020).

After entering the 21st century, leptospirosis has been controlled at a pretty low prevalence in China (Zhang et al., 2012). Taking Hubei province for example, the final wave of leptospirosis outbreak occurred in 1996, and since then, the leptospirosis maintained a sporadic level (Figure 1) (Ma et al., 2021). In this context, if the patients with MMD were originated from leptospiral exposure, then their age at onset (AAO) might have increased over time as a reduced exposure to leptospiral infection since 1996.

In this study, we performed a multicenter, retrospective epidemiologic survey in a large real‐world sample, to explore whether the AAO increased over the last 25 years (1996–2020) in Chinese patients with MMD.

## 2 METHODS

We analyzed data of patients diagnosed with MMD according to different criteria available over time in 1997, 2012, and 2021 revised version (Fukui, 1997; Hashimoto et al., 2012; Kuroda et al., 2022), and regularly attending four tertiary hospitals from 1996 to 2020 in Hubei, China (Wuhan General Hospital of Guangzhou Military Command; Zhongnan Hospital of Wuhan University; Tongji Medical College, Huazhong University of science and technology; Xiangyang Central Hospital).

Data were gathered and recorded for each patient upon the initial visit, including AAO; birth date; sex; year of diagnosis; home address. That address was unclear, or outside the Hubei province was excluded, as the leptospirosis epidemic history was not completely consistent among provinces. Early‐onset (EO), intermediate‐onset (IO), and late‐onset (LO) were defined by disease onset at age before 20 years of age, from 20 to 49 years, and at 50 years or over, respectively.

The AAO and birth year distributions were categorized into 5‐year intervals, and the proportion in each group was displayed on the heatmap. All analyses were stratified by MMD diagnosis time and categorized into five periods according to the calendar year: 1996–2000, 2001–2005, 2006–2010, 2011–2015, and 2016–2020. The following comparison analyses were then done across each period: (1) median (IQR) AAO; (2) female‐to‐male sex ratio; and (3) proportions with onset at different age classes.

The average AAO was shown as the median and interquartile range (IQR) for nonnormal distribution. Statistical analyses were performed using SPSS statistical software (version 23.0, IBM Corp, Armonk, NY, 2014), and graphics were prepared using GraphPad Prism software (GraphPad Prism version 8.00, San Diego, CA, USA). Temporal tendencies over periods were analyzed by the Jonckheere–Terpstra test and the χ^2^ test for trends for continuous and categorical variables, respectively.

## 3 RESULTS

Out of 2182 regularly attending our centers, we excluded 86 patients for unclear addresses and 238 patients for out of the study area. Finally, we included 1858 patients in this study, with 878 women and 980 men. The female‐to‐male sex ratio was stable over time at 0.90 (95% CI 0.81–0.97, *p* = .287), without a female predominance. Their median (IQR) AAO was 47 (39–55) years, ranging from 2 to 83 years. The detailed trends in demographic features of MMD were described in Table 1.

**TABLE 1:** The detailed trends in demographic features of MMD from 1996 to 2020

	1996–2000 (*n* = 27)	2001–2005 (*n* = 67)	2006–2010 (*n* = 287)	2011–2015 (*n* = 570)	2016–2020 (*n* = 907)	Total (*n* = 1858)
Female, no. (%)	13 (48.1)	29 (43.3)	141 (49.1)	287 (50.4)	408 (45.0)	878 (47.3)
Sex ratio (F/M)	0.93	0.76	0.97	1.01	0.82	0.90
AAO, median (IQR)	26 (14–37)	35 (31–41)	41 (41–48)	46 (40–53)	51 (43–57)	47 (39–55)
EO, no. (%)	9 (33.3)	7 (10.4)	5 (1.7)	7 (1.2)	18 (2.0)	46 (2.5)
IO, no. (%)	16 (59.3)	57 (85.1)	228 (79.4)	363 (63.7)	395 (43.6)	1059 (57.0)
LO, no. (%)	2 (7.4)	3 (4.5)	54 (18.8)	200 (35.1)	494 (54.5)	753 (40.5)

MMD (moyamoya disease); F/M (female to male); AAO (age at onset); IQR (interquartile range); EO (early‐onset, < 20 years); IO (intermediate‐onset, 20–49 years); LO (late‐onset, ≥ 50 years).

**FIGURE 2:**
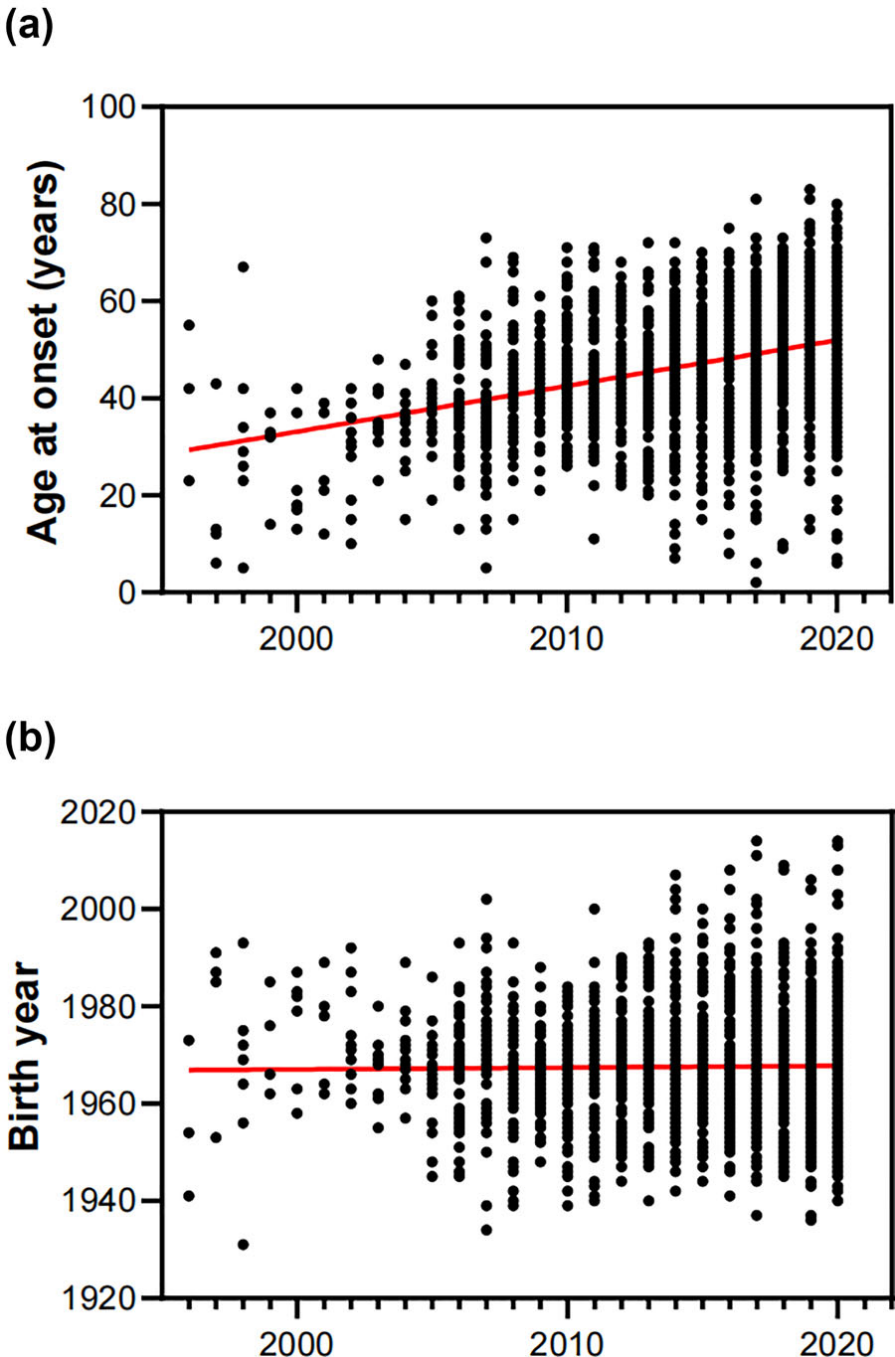
Trends in AAO (a) and birth year (b) of MMD cases over time by Spearman's rank correlation (red line).

**FIGURE 3:**
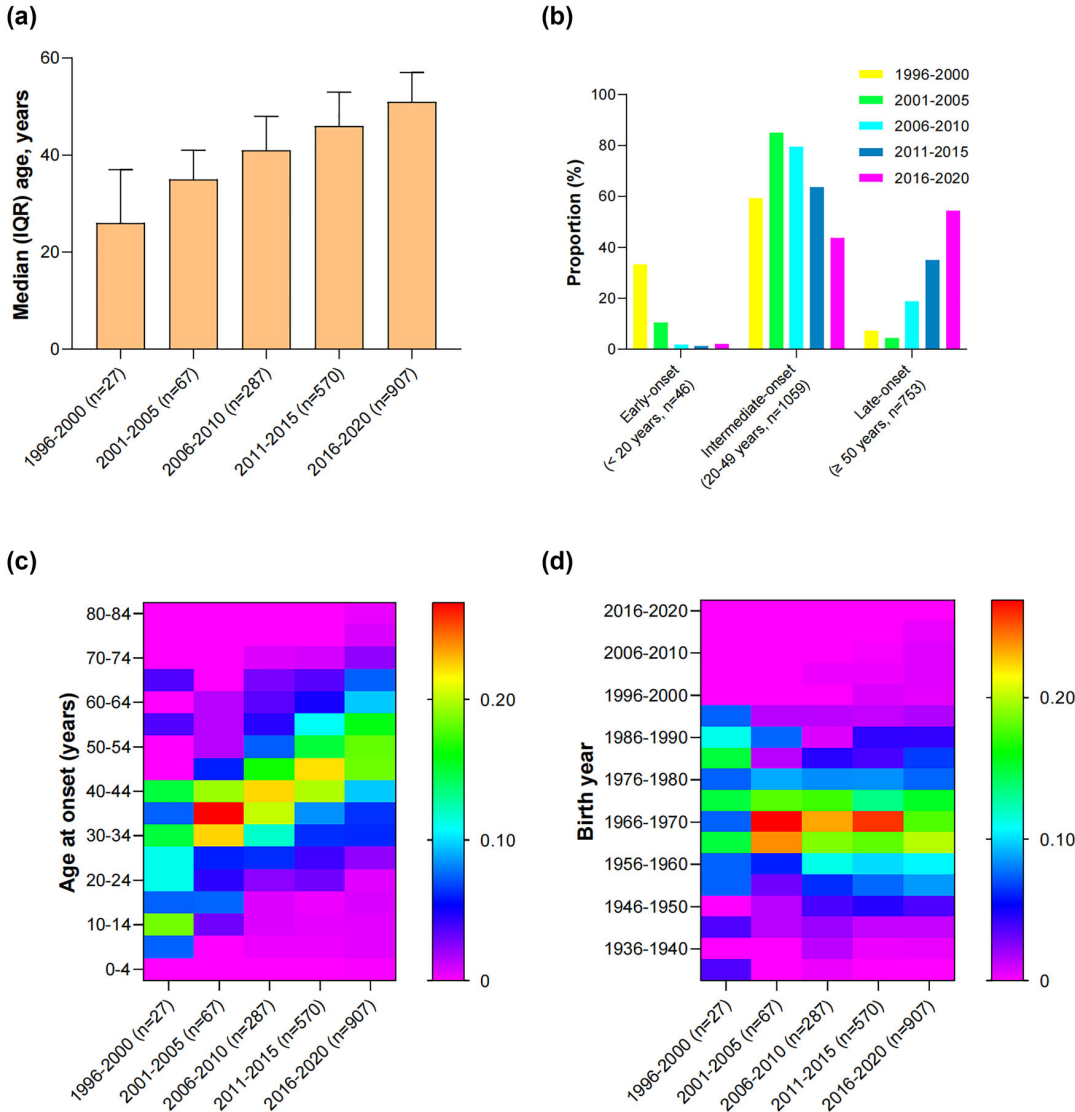
Temporal trends in median AAO (a) and classes of AAO (b). Trends in AAO (c) and birth year (d) on heatmap at every 5‐year interval.

The AAO increased steadily from 1996 to 2020, yet cases from all years exhibit similar dependence on birth year. Spearman's rank correlation showed case age significantly increased at the rate of 0.94 years per year (*r* = 0.406, *p* < .0001), while no trend was observed in case birth years through time (*p* = .512) (Figures 2a and 2b). Importantly, the median (IQR) AAO has increased significantly from 26 (14–37) years (1996–2000) to 51 (43–57) years (2016–2020) (*p* < .0001) (Figure 3a).

The EO and IO MMD showed a decreased trend, while a reverse trend was observed among LO MMD over time (*p* < .0001) (Figure 3b). Despite only 2.5% (46/1858) EO MMD was observed, the binomial test suggested this proportion was significantly higher in 1996–2000 (33.3%, *p* < .0001) and 2001–2005 (10.4%, *p* < .001). In addition, the AAO shows an aging trend that the proportion of LO MMD has dramatically gone from 4.5% (2001–2005) to 54.5% (2016–2020) (*p* < .0001).

From the heatmap, it was apparent that the AAO shifted toward older over time (Figure 3c). In contrast, the people born between 1961 and 1975, growing up during leptospirosis outbreaks, were stably susceptible to MMD, accounting for 55.9% (1038/1858) of the sample. Besides, the birth cohorts after 1996, with a rapidly reduced leptospiral exposure, rarely got involved in MMD, no matter in which period (Figure 3d). So, on average, the specific birth cohorts remained at high risk of MMD, even as members got delayed diagnoses over time, leading to the increasing trend of AAO.

## 4 DISCUSSION

Through a 25‐year multicenter retrospective analysis, this study firstly observed a significantly increased AAO for Chinese patients with MMD at the rate of 0.94 years per year. The median AAO of MMD has increased from 26 to 51 years from 1996–2000 to 2016–2020, showing an aging trend. The EO and IO MMD showed a decreased trend, while a reverse trend was observed among LO MMD over time. In addition, the birth year changed minimally over time, suggesting a birth cohort effect might exist. The role of leptospiral exposure on the birth cohort effect was discussed.

This study demonstrated that the AAO shifted toward older over time for Chinese MMD. Similar trends of change were reported in other regions. Kim et al. reported the mean age of MMD patients was 33.5 in 2005 and increased to 42.5 in 2013 for Korean patients (Kim et al., 2015). One of the possible causes of the increase in AAO is the rising life expectancy observed in the general population. However, this study reported that AAO among patients with MMD significantly increased at a rate of 0.94 years per year, which obviously exceeds the rate of increases in the age in the general population. Therefore, general population aging cannot fully explain the increasing AAO.

The increased AAO might reflect a birth cohort effect, with members got delayed diagnosis at different time. This study found no trend in case birth years over time, and the 1961–1975 birth cohorts were mostly susceptible, while those born after 1996 rarely got involved. The birth cohort effect might be explained by a complex interplay with environmental risk factors changing over time. This study demonstrated the susceptible birth cohorts were growing up during leptospiral exposure increased in the 1970–1980s. While when the leptospiral exposure reduced since 1996, the subsequent birth cohorts rarely got involved in MMD. Although lack of direct evidence, these findings suggested changes in leptospiral exposure might be a considerable environmental factor to shape the birth cohort effect of MMD. As a result, the affected birth cohorts were diagnosed at different time, which led to the increasing trend of AAO over time.

The age distribution of MMD is not always a bimodal peak pattern. This study observed a decreased EO MMD and an upward trend for LO over time. Similar trend has been reported in previous incidence studies. Chen et al. (2014) found adults exhibited an upward trend in incidence while children had a decreased incidence in Taiwan. Baba et al. (2008) reported that the highest peak of incidence shifted from children to adults in Japan. These studies emerge with an aging trend for MMD. One possible reason might be the declining birth rate in the 21st century, and another might be the reduced exposure to leptospiral infection for children. However, exploring the reasons goes beyond our objectives, and requires a different study design. We would instead point out a standard treatment needs to be constructed for LO MMD.

There are two limitations in this study. On one hand, the data in present study was from Hubei province, data from other provinces was absent. However, most provinces had a similar history of leptospirosis epidemic, hence we believe in our results are quite representative for China. On the other hand, the hypothesized causal link between leptospiral exposure and MMD was based on whether the susceptible birth cohorts were living in the leptospirosis outbreaks, which was lack of direct evidence.

In conclusion, this study has clearly demonstrated that the AAO in Chinese patients with MMD has increased significantly over the last 25 years. The increased AAO reflected a birth cohort effect, which was associated with changing environmental factors, and leptospiral exposure might be a potential one.

### PEER REVIEW

The peer review history for this article is available at https://publons.com/publon/10.1002/brb3.3034.

## Data Availability

The data that support the findings of this study are available from the corresponding author upon reasonable request.
